# The House of Steinway

**Published:** 1874-12

**Authors:** 


### THE HOUSE OF STEINWAY.

We remarked last month that by the simple accident of the exhibition of a piano in the New York Crystal Palace The House of Steinway became famous in a day. It may interest our readers to learn the details of an accident which bestowed upon America the credit of furnishing the finest musical instruments in the world.

Henry Steinway, the founder of the great house of Steinway & Sons, was a native of Brunswick, Germany. As a boy he was apprenticed to a cabinet maker, but was drawn away, by natural bias, from the business he had learned to the making of organs and pianos. He amused his boyhood by repairing old musical instruments and making new ones. He made a cithara and a guitar for himself before he was fourteen years old, with only such imperfect tools as a boy commands. After he had served out his time he entered steadily upon the occupation to which he had taken such a fancy during his whole life. For many years he was a German piano-maker, producing, in the slow, German manner, two or three excellent instruments a month; striving ever after higher excellence, and growing more and more dissatisfied with the limited sphere in which the inhabitant of a small German State necessarily works. In 1849, being then over fifty years of age, and the father of four intelligent and gifted sons, he looked to America for a wider range for his genius, and a more promising home for his boys. With German prudence he sent one of his sons to New York to see what prospect there might be for another maker of pianos. Charles Steinway came, saw, approved, returned, reported; and in 1850 all the family arrived in New York, except the eldest son, Theodore, who continued the piano business, already well established in Brunswick. The father again manifested his wisdom, by not going immediately into business. He appreciated the fact, that the art of making a good piano and the art of selling it were quite distinct. He thought it advisable to study the trade from the American stand-point before embarking in it. So he deposited the small capital which he brought from Germany, put on the journeyman's apron, and worked for three years in a New York piano factory. He kept his eyes open, and grew in knowledge day by day. So also did his sons, who were all employed in a similar way—one of them, fortunately, becoming a tuner, which brought him into relations with many music teachers. During these three years, their knowledge and their capital increased every day, for they lived as wise men in such circumstances do live, who mean to control their destiny. In short, they kept their eyes open and lived on half their income. In 1853, in a small back shop in Varick street, with infinite pains, they made their first American piano, and a number of teachers and amateurs were invited to listen to it. It was warmly approved and speedily sold. Ten men were then employed, who produced for the next two years one piano a week. In 1855, the Messrs. Steinway, still but little known to the public, placed one of their pianos from their shop in the New York Crystal Palace Exhibition. Of course the judges who were elected to decide upon the merits of the various instruments exhibited had to look at this obscure intruder among the rest. A member of that committee has recorded the scene which occurred when they reached the unknown competitor :

"They were pursuing their rounds, and performing their duties with an ease and facility that promised a speedy termination to their labors, when suddenly they came upon an instrument that, from its external appearance—solidly rich, yet free from the frippery that was then in fashion—attracted their attention. One of the company opened the case and carelessly struck a few cords. The others were doing the same with their neighbors, but somehow they ceased to chatter when the other instrument began to speak. One by one the jurors gathered around the strange polyphonist, and, without a word being spoken, every one knew it was the best piano-forte in the Exhibition. The jurors were true to their duties. It is possible that some of them had predilections in favor of other makers; it is certain that one of them had—the writer of the present notice. But when the time for the award came, there was no argument, no discussion, no base presentment of minor claims; nothing, in fact, but a hearty endorsement of the singular merits of the strange instrument."

From that time the course of The House of Steinway was onward and upward. The tide of California gold was flowing in, and every day some one was getting rich enough to treat his family to a new piano. It was the Messrs. Steinway who chiefly supplied the new demand, without lessening by one instrument a month the business of the older houses. Within themselves they combined the education and talent and business tact requisite for conducting the business. Up to nearly the close of an honored and useful life, the father of the family, in the dress of a workman, attended daily at the great factory, as vigilant and active as ever, even after reaching the age of threescore and ten; while his surviving sons were as busily engaged in their several departments as they were in the infancy of the establishment.

In our next issue we will try to give our readers an insight into the methods employed in constructing a piano, and the materials entering into its composition.

